# 507. Trajectories of Newly Initiated Pre-Exposure Prophylaxis (PrEP) Use among Priority Populations with Unmet Needs for PrEP in the USA

**DOI:** 10.1093/ofid/ofae631.159

**Published:** 2025-01-29

**Authors:** Li Tao, Juan Yang, Joshua Gruber, Chris Nguyen, Woodie Zachry

**Affiliations:** Gilead Sciences, Foster City, CA; Gilead Sciences, Foster City, CA; Gilead Sciences, Foster City, CA; Gilead Sciences, Inc., Foster City, California; Gilead Sciences Inc, Foster City, California

## Abstract

**Background:**

PrEP is an important strategy in HIV-1 prevention, yet disparities persist in its uptake across diverse demographic groups. This study aims to identify priority populations with unmet PrEP needs and to describe new PrEP prescription initiation patterns in these groups.

**Figure 1.**
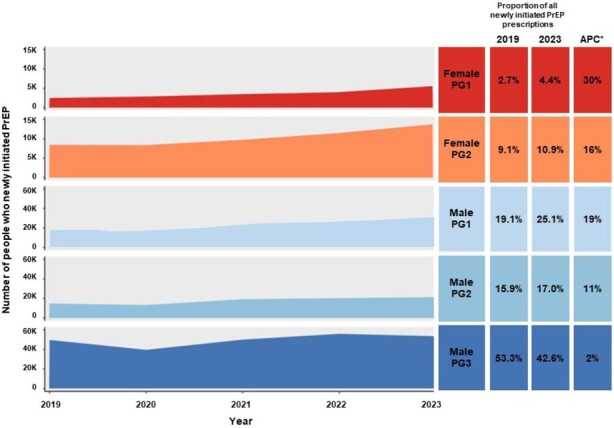
Trajectories for Number of Individuals Who Newly Initiated PrEP between 2019 and 2023 by Priority Groups in the United States *APC from 2019 to 2023.

HIV–1 risk factors on record in the claims dataset were a group of ICD-based diagnosis and CPT codes that were highly recommended for PrEP use by providers, including any diagnoses of any sexually transmitted diseases (A50-64), contact with and exposure to communicable diseases (Z20.6, Z20.2, Z20.828, Z20.9), high-risk sexual behavior (Z72.51-Z72.53), other hazardous exposures (Z77.21, Z77.9), contact with hypodermic needle (W46.0, W46.1), long-term prophylaxis (Z79.899), HIV prevention counseling (99401-99404), and HIV screening procedures (86689, 86701-86703, 87389, 87534-87538, G0432-G0435).

Female PG1: Females without recorded HIV-1 risk factors; Female PG2: Females with recorded HIV-1 risk factors; Male PG1: Males without recorded HIV-1 risk factors; Male PG2: (1) Males with recorded HIV-1 risk factors and had Medicaid coverage; (2) Males with recorded HIV-1 risk factors, with commercial insurance (including ∼23% assistance programs, Medicare, and other mixed/missing on plan coverage) and residing in Black (non-Hispanic) or Hispanic neighborhoods; Male PG3: Males with any recorded HIV-1 risk factors, with commercial insurance (including ∼23% assistance programs, Medicare, and other mixed/missing on plan coverage) and residing in White (Non-Hispanic White, and with ∼24% in non-Hispanic Asian or other mixed/missing) neighborhoods.

APC, annual percent change; CPT, Current Procedural Terminology; ICD; International Classification of Diseases; PG, priority group; PrEP, pre-exposure prophylaxis.

**Methods:**

Data on PrEP prescriptions and new HIV-1 diagnoses (2019–2023) were obtained from an IQVIA claims database. PrEP-to-Need Ratio (PNR; number of individuals using PrEP in a year divided by new HIV diagnoses in the previous year) was calculated for subpopulations defined by five PNR-associated factors: sex, insurance, recorded HIV-1 risk factors (identified by diagnosis or procedure codes), ‘*Ending the HIV Epidemic*’ jurisdictions, and neighborhood race/ethnicity composition. Lower PNR indicates higher unmet need. Using cluster analysis, subpopulations with similar 2019 PNR values were classified into five population priority groups (PGs). Trends of newly initiated PrEP prescriptions and its annual percent change (APC) were assessed by PG, and PNR values for PGs in 2019 and 2023 were compared.

**Figure 2.**
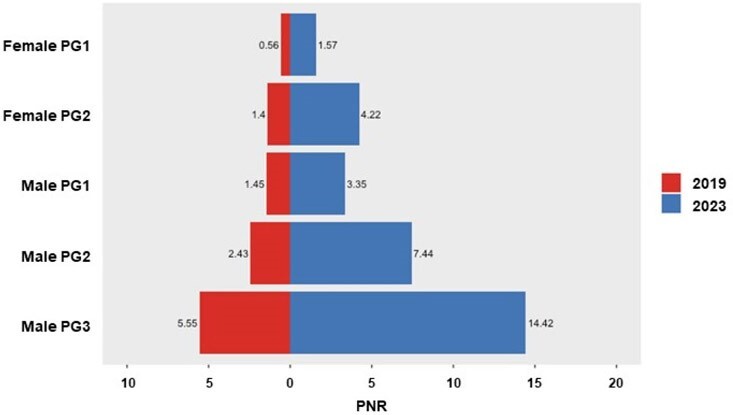
PrEP-to-Need Ratio in 2019 and 2023 by Priority Groups in the United States Lower PNR indicates higher unmet need. PG, priority group; PNR, PrEP-to-Need Ratio; PrEP, pre-exposure prophylaxis.

**Results:**

A large proportion of newly initiated PrEP users in both 2019 (53% of 93,344) and 2023 (43% of 125,819) were males with HIV-1 risk factors, using commercial insurance, and residing in White dominant neighborhoods; this group had a marginal increase in PrEP initiation (APC 2%; Male PG3, **Figure 1**) and the lowest unmet needs (**Figure 2**). Other males with HIV-1 risk factors (Male PG2; those commercially insured residing in Black/Hispanic neighborhoods or those on Medicaid across all neighborhoods) had a moderate increase in PrEP initiation (APC 11%) and higher unmet needs. Females and males without recorded HIV-1 risk factors showed the highest PrEP initiation increase (Female PG1, APC 30%, and Male PG1, APC 19%), but retained the most substantial unmet needs from 2019 to 2023.

**Conclusion:**

This study identified individuals without known HIV-1 risk factors and with a high rate of new PrEP initiation; this highlights an ongoing unmet need for PrEP in this population. Individuals on Medicaid or residing in Black/Hispanic communities would benefit from increased PrEP uptake to address disparities in HIV prevention. These findings emphasize the need to ensure equitable PrEP access across diverse populations in the real world.

**Disclosures:**

**Li Tao, MD, PhD**, Gilead Sciences, Inc.: Employee|Gilead Sciences, Inc.: Stocks/Bonds (Public Company) **Juan Yang, PhD**, Gilead Sciences, Inc.: Employee|Gilead Sciences, Inc.: Stocks/Bonds (Public Company) **Joshua Gruber, PhD MPH**, Gilead Sciences, Inc.: Employee|Gilead Sciences, Inc.: Stocks/Bonds (Public Company) **Chris Nguyen, PharmD**, Gilead Sciences, Inc.: Employee|Gilead Sciences, Inc.: Stocks/Bonds (Public Company) **Woodie Zachry, RPh, PhD**, Gilead Sciences, Inc.: Employee|Gilead Sciences, Inc.: Stocks/Bonds (Public Company)

